# Don't be perplexed by the plexus! A practical approach to brachial plexus ultrasound

**DOI:** 10.1093/bjro/tzaf003

**Published:** 2025-03-19

**Authors:** James F Griffith

**Affiliations:** Department of Imaging and Interventional Radiology, The Chinese University of Hong Kong, Shatin NT, Hong Kong SAR, China

**Keywords:** ultrasound, brachial plexus, technique, pathology, irradiation plexopathy, tumour, trauma

## Abstract

Ultrasound is as accurate as MRI in the detection of most brachial pathologies but tends to be underutilized in clinical practice compared to MRI. The main reason for this under-usage is a relative lack of knowledge regarding how to perform brachial plexus ultrasound and a lack of awareness of the ultrasound appearances of brachial pathologies. This review serves to re-address this imbalance by providing a practical overview on how to perform brachial plexus ultrasound as well as highlighting the ultrasound appearances of common pathologies likely to be encountered in everyday clinical practice.

## General considerations

The brachial plexus is the neural highway to the upper limb. It extends from the ventral rami of the cervicodorsal roots to the terminal branches in the axilla. The brachial plexus is amiable to ultrasound examination in most patients.

### Limitations of brachial plexus ultrasound

Although the intra-foraminal or intraspinal portions of the nerve roots are not assessable to ultrasound examination, these are not, in essence, part of the brachial plexus, which starts at the level of the ventral rami. Also, a small segment of the brachial plexus beneath the clavicle (ie, the “costoclavicular gap”) is not assessable to ultrasound examination, though the brachial plexus immediately proximal and distal to this short area can be consistently examined. Also, dynamic spectral Doppler ultrasound can still allow one to assess vascular compression in the costoclavicular gap although the compressive site may not be directly visible. A very small number of patients may not be suitable for ultrasound examination due to the presence of an open wound or severe soft tissue swelling around the brachial plexus. Also, a small number of patients cannot fully abduct the arm, limiting ultrasound examination of the axillary region.

### Benefits of brachial plexus ultrasound

As clinical localization of symptoms to the brachial plexus is difficult, ultrasound is a good means of screening for brachial plexus pathology. A negative ultrasound examination effectively excludes most brachial plexus pathology. The likelihood of finding an abnormality on MRI when ultrasound examination is negative is extremely low.^1–3^ Ultrasound is as accurate as MRI in the detection of brachial plexus pathology.^1^^,^^2^ Ultrasound can be performed relatively quickly compared to MRI and allows ready comparison of both sides. Also, high-quality ultrasound of the brachial plexus is not usually affected by body habitus like other body areas, such as abdominal ultrasound. Standard ultrasound of the brachial plexus, which involves examination of both sides, can be completed in 15 min. The cost is similar to other neck ultrasound examinations. Ultrasound for suspected thoracic outlet syndrome (TOS) can take 45 min to complete.

Several plexal disorders, such as neuralgic amyotrophy or inflammatory neuropathy, are not confined to the plexus and may also involve extraplexal nerves either in isolation or together with plexal involvement. In this regard, ultrasound enables ready examination of nerves beyond the brachial plexus. Ultrasound, in addition, can facilitate ultrasound-guided biopsy for confirmation of suspicious findings or ultrasound-guided therapeutic injection. It should be remembered that almost two-thirds of ultrasound and MRI examinations of the brachial plexus will be normal. This is a consistent finding even in centres where expertise in brachial plexus imaging exists.^1^^,^^2^ This most likely reflects difficulty with localizing symptoms to the brachial plexus rather than a limitation of the ultrasound or MRI technique per se. One should not become perturbed if one does not uncover brachial plexus pathology in most clinical situations.

Finally, do not be perplexed by the plexus! Remember that there are 5 roots, 3 trunks, 6 divisions, 3 cords, and 5 terminal branches. Finding these different components on ultrasound examination is not difficult. Plexal anatomy varies considerably from 1 patient to the next, though there is very good side-to-side symmetry.

## Anatomy of the brachial plexus

The brachial plexus is formed from the C5-T1 ventral rami. These 5 ventral rami combine to form 3 trunks, 6 divisions, 3 cords, and 5 terminal branches. This classic pattern, however, is not present in most patients. One cannot rely on having a definite number of trunks, divisions, or cords at any location.

The C5 and C6 ventral rami pass between the anterior and posterior tubercles of the C5 and C6 cervical transverse processes, respectively. The C7 transverse process has either an absent or rudimentary anterior tubercle so that the C7 root lies anterior to the posterior tubercle of C7. The C8 and T1 roots lie more deeply. The normal cross-sectional area (CSA) of the C5 root is about 6 mm^2^, C6 is 9 mm^2^, and C7 and C8 are about 11 mm^2^, though there is 30%-40% difference in reported CSA values,^4^ probably reflecting difficulties in demarcating the root outline and the location at which the nerve root is measured.

The ventral rami pass in the “interscalene gap” or “interscalene triangle” between the scalenus anterior muscle anteromedially and the scalenus muscle posterolaterally. Here the roots unite to form the upper, middle, and lower trunks. The average CSA of the upper trunk is about 17 mm^2^, while that of the middle and lower trunks is about 14 mm.^2.5^ The trunks have only been measured in one study, probably reflecting the fact that the trunks are not always visible as a definable structure, with the roots either quickly becoming divisions or the upper roots passing through or anterior to the scalenus anterior muscle. Great variability exists across the population as regards where the roots pass and where the trunks form and divide. Although anatomical variability of the roots, trunks, divisions, and, to a lesser degree, the cords is quite frequent, the main thing to realize is that while there is considerable variability between patients, there seems to be little side-to-side variability within the same patient.

The C5 and C6 roots or the upper trunk commonly pass either through or anterior to the anterior scalenus muscle. The dorsal scapular artery often passes posteriorly between the upper and middle trunks. The suprascapular nerve emerges from the C5 root of the upper trunk of the brachial plexus in the interscalene region. The suprascapular nerve can be traced more distally as it separates from the divisions to run laterally deep to the omohyoid muscle. The omohyoid muscle attaches to the upper margin of the scapular blade just lateral to the suprascapular notch and is a good marker for the suprascapular nerve, which runs from the extrascalene region to the scapula in a similar direction.

Beyond the scalene muscles (in the “extrascalene region”), the trunks divide into anterior and posterior divisions in the supraclavicular fossa. Three trunks give rise to 6 divisions. These divisions align laterally and superolaterally to the subclavian artery. The anterior divisions innervate the (anteriorly located) flexor muscles of the upper limb, while the posterior divisions innervate the (posteriorly located) extensor muscles. The transverse cervical artery often loops over the divisions in the supraclavicular fossa. The “costoclavicular gap,” which is located between the clavicle and the first rib, is the narrowest part of the brachial plexus passageway. It is also probably the most common site of subclavian arterial or venous compression.

The subclavian artery becomes the axillary artery on passing over the first rib within the costoclavicular gap. Beyond the costoclavicular gap, the 6 divisions form the 3 cords in the “subpectoralis minor space” (or “subcoracoid space”). The cords are classically located lateral, medial, and posterior to the axillary artery. The lateral and medial cords innervate the flexor muscles, while the posterior cord innervates the extensor muscles.

The cords pass into the axilla, where they give rise to the terminal branches. The lateral cord gives rise to the median and musculocutaneous nerves. The medial cord gives rise to the median and ulnar nerves. The posterior cord gives rise to the axillary and radial nerves as well as some smaller branches. The nerves are arranged around the axillary artery in a quite consistent position. With the arm abducted, the median nerve lies at 10 to 11:00 in a superolateral position, the ulnar nerve lies at 1 to 2:00 in a superomedial position, while the radial nerve lies at 4 to 6:00 in an inferomedial position. The musculocutaneous nerve usually runs between the coracobrachialis and biceps muscles before piercing the coracobrachialis muscle.

## Ultrasound technique

**Figure 1.**
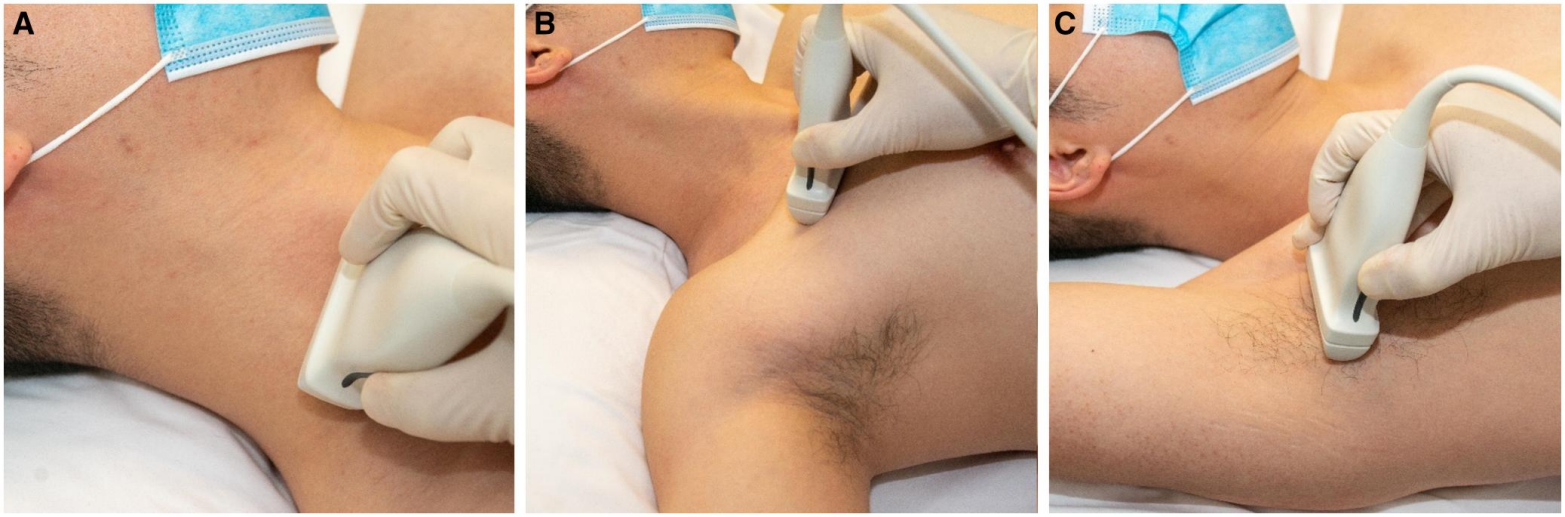
Scan position for the (A) supraclavicular brachial plexus, (B) subcoracoid area, and (C) axillary fossa.

The patient should lie supine on the examination table with a gown tied behind the upper back region so that both shoulders are fully exposed and the arms can be fully abducted. The brachial plexus components are best examined almost solely in the transverse plane (Figure 1). Longitudinal scanning can be helpful in avulsion or traction injury, though for the most part, longitudinal scanning is not necessary. A high-end ultrasound machine with a high-resolution 12-5 MHz linear array probe should be used to obtain optimal image quality. Lower resolution curvilinear probes are not required. A small 7-15 MHz high-resolution “hockey-stick” probe can be helpful when examining infants or young children with small, short necks. It can also help in providing finer detail of previously identified pathologies, though it rarely leads to any change in diagnosis.

Ultrasound of the brachial plexus is a standardized examination wherein all parts of the brachial plexus should be routinely assessed. The roots, trunks, and divisions are monofascicular and hypoechoic, while the cords and terminal branches are more polyfascicular and hyperechoic. It is imperative to examine both sides of the brachial plexus, starting with the unaffected or least affected side. This is a key part of the examination for, while considerable population variability in brachial plexus morphology exists, there is relatively little intrinsic side-to-side variability. The contralateral normal or less affected side acts as a good internal reference as to what the brachial plexus on the affected side is expected to look like.

**Figure 2.**
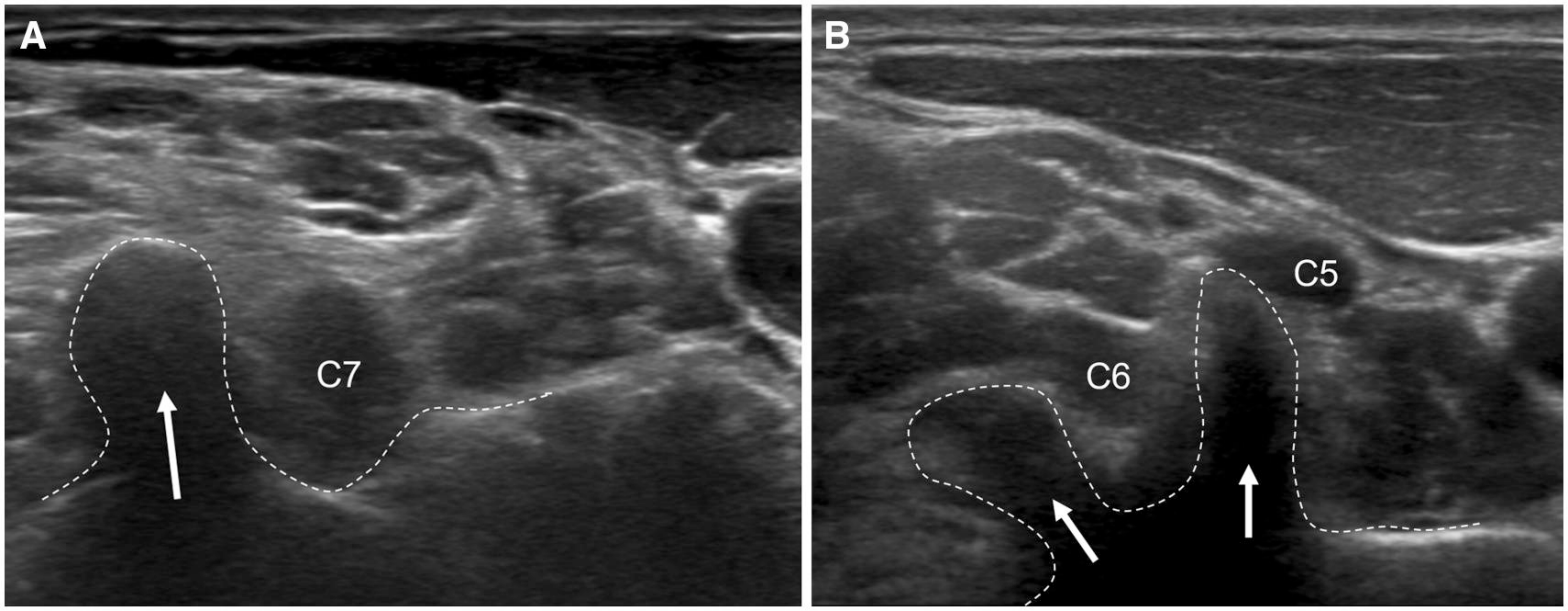
(A) The C7 transverse process has a large posterior tubercle (arrow) (“thumbs up sign”) with no anterior tubercle. The C7 root lies anterior to the posterior tubercle. (B) The C6 vertebral body has equal-sized anterior and posterior tubercles. The C6 nerve root lies between the C6 tubercles.

It is best to start the examination in the interscalene region, as this part of the brachial plexus is most readily visualized. The upper, middle, and lower trunks can be seen as the “traffic light sign,” though often. The classic traffic light sign will not be visible (Figure 2). The amount and compressibility of the fat surrounding the trunks in the interscalene region should be noted.

**Figure 3.**
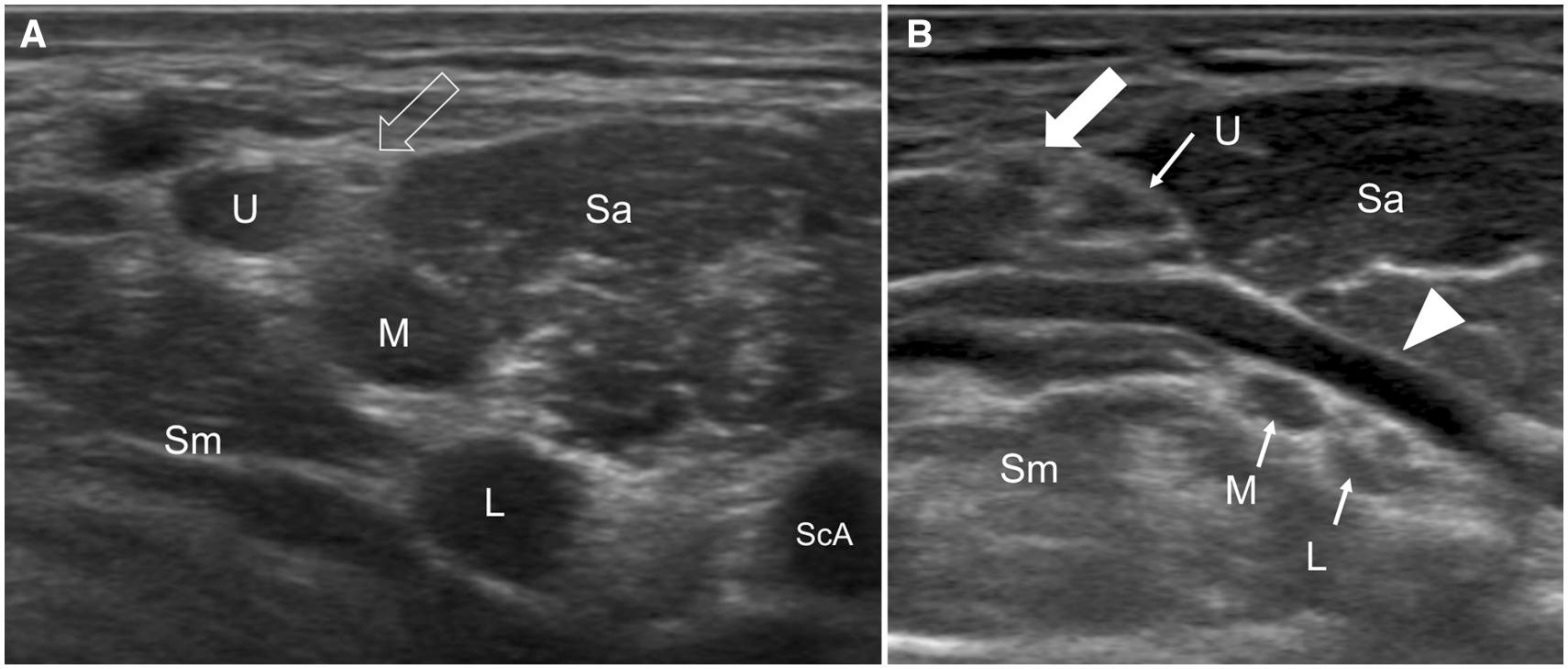
(A) Transverse ultrasound of the interscalene gap shows the monofascicular upper (U), middle (M), and lower (L) trunks of the brachial plexus. The classic “traffic light sign” is not always seen. The phrenic nerve is shown (open arrow). (B) Transverse ultrasound of the interscalene gap shows the dorsal scapular artery (arrowhead) passing through the middle and upper trunks of the brachial plexus. The suprascapular nerve is shown (block arrow). Abbreviations: Sa = scalenus anterior muscle; Sm = scalenus medius muscle.

The transducer can then be moved proximally in the same transverse plane to examine the individual roots emerging from the cervical spine. The C7 root is a good marker (“thumbs up sign”) (Figure 3). Once the C7 root is identified, move cephalad to examine the C6 root between the tubercles of the C6 transverse process (“V-sign”). The C5 root has a similar appearance, though with a narrowed distance between the anterior and posterior tubercles. Moving caudad from C7, one would visualize the C8 and occasionally the T1 roots.

Pay particular attention to any undue swelling, distortion, indentation, or abrupt angulation of the lower roots or trunk, as this may indicate neural compression in TOS.^6–8^

Returning to the interscalene region, observe the suprascapular nerve passing posteriorly from the C5 root of the upper trunk to lie on the surface of the scalenus medius muscle and deep to the omohyoid muscle. The omohyoid muscle can be followed as a marker for the suprascapular nerve down to this insertion near the suprascapular notch. Note the size of the suprascapular nerve. Focal enlargement of the suprascapular nerve anywhere along its course can be a sign of neuralgic amyotrophy.

**Figure 4.**
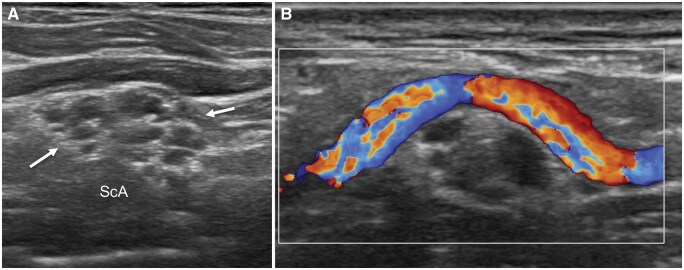
(A) Transverse ultrasound of the posterior triangle showing the “bunch of grapes” appearance of the divisions (arrows) aligned superomedial to the subclavian artery (ScA). (B) Transverse colour Doppler ultrasound shows the transverse cervical artery passing over the divisions of the brachial plexus.

**Figure 5.**
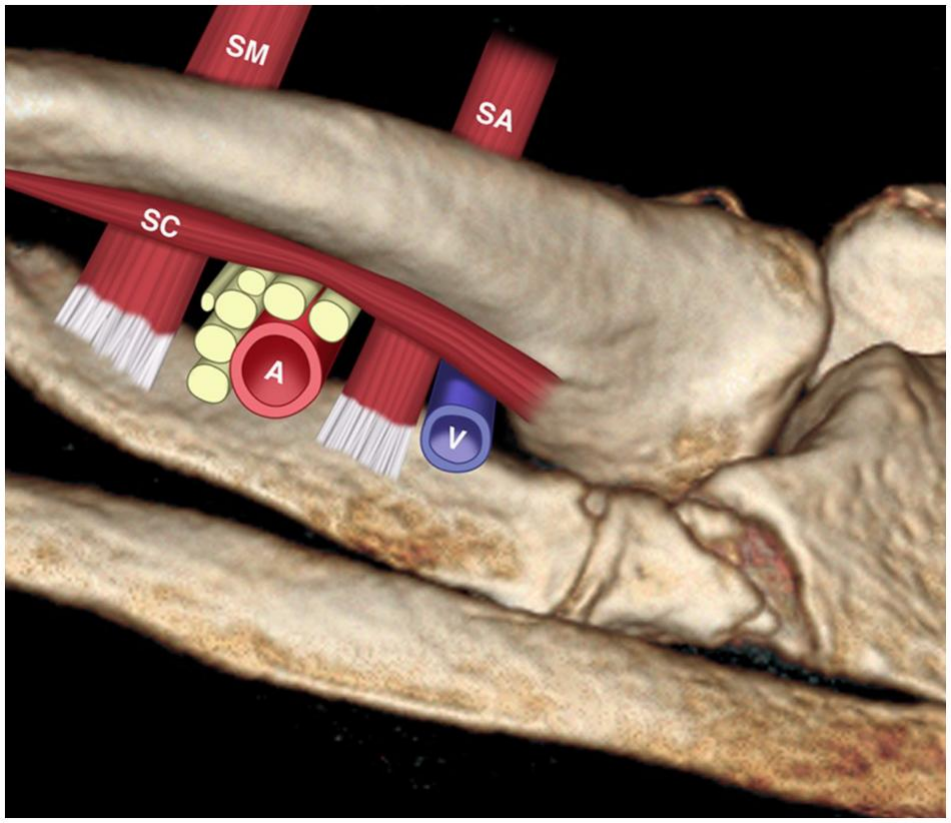
Schematic diagram of costoclavicular gap. Abbreviations: SC = subclavius muscle; SA = scalenus anterior; SM = scalenus medius; A = subclavian artery; V = subclavian vein.

Scanning in the same transverse plane more lateral to the scalene muscles, one will appreciate the divisions of the brachial plexus lining up lateral, superolateral, or superior to the subclavian artery. This has been likened to a “bunch of grapes” (Figure 4). Similar to the interscalene region, make note of the amount and appearance of the fat around the divisions as well as the apparent stiffness of this fat, as perineural fibrosis is an indicator of radiation-induced plexopathy. Follow the divisions in a transverse plane, angling the transducer down behind the clavicle to follow the divisions to the costoclavicular gap (Figure 5).

**Figure 6.**
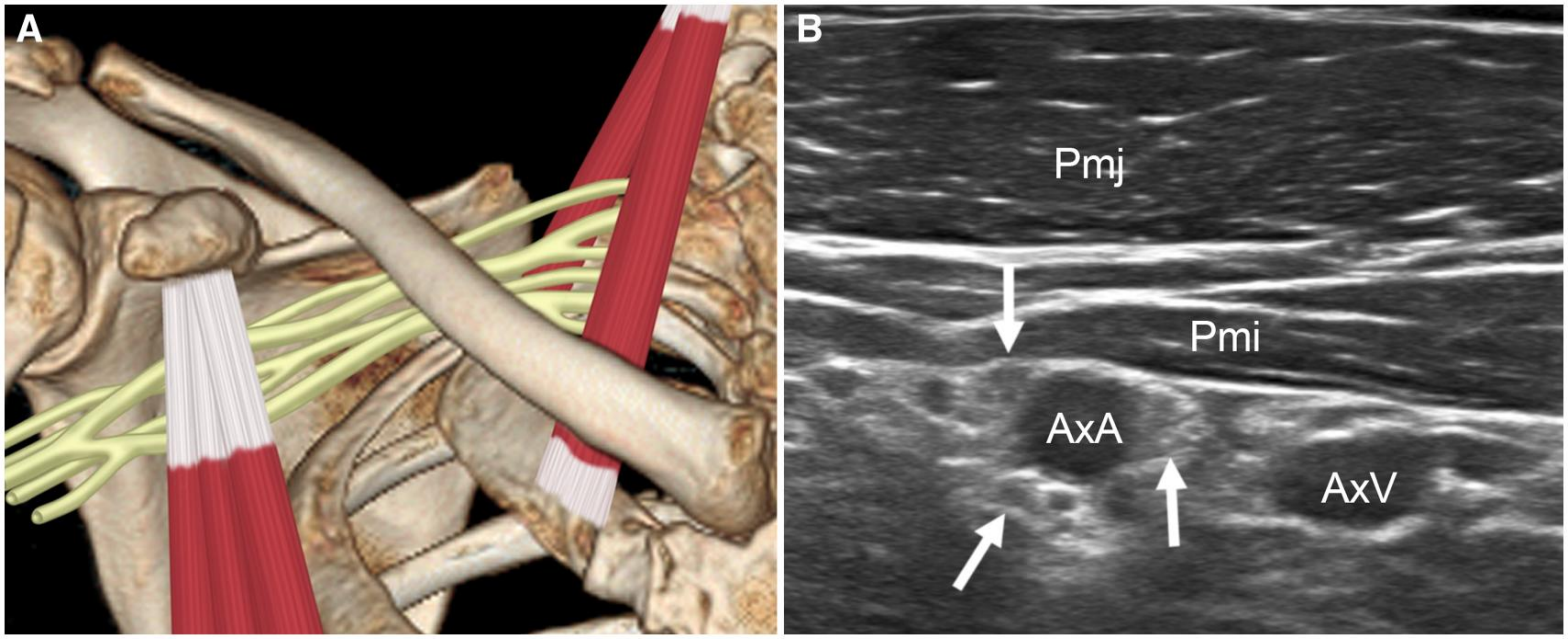
(A) Schematic diagram of infraclavicular area. (B) Transverse ultrasound examination showing the cords (arrows) aligned alongside the axillary artery (AxA). Abbreviations: Pmi = pectoralis minor; Pmj = pectoralis major; AxV = axillary vein.

Then, abduct the arm to 90° and place the transducer immediately below the clavicle. The divisions will align in the same location around the axillary artery as they were in the supraclavicular region. The divisions will then merge into 3 cords typically located laterally, posteriorly, and medial to the axillary artery (Figure 6). The subpectoral space is the most difficult part of the brachial plexus to visualize. If a cord or cords cannot be definitively identified, one can still inspect the tissues around the axillary artery, making sure that there is no undue cord swelling, which would make the cord more conspicuous, or any mass lesion in the region of the cords.

**Figure 7.**
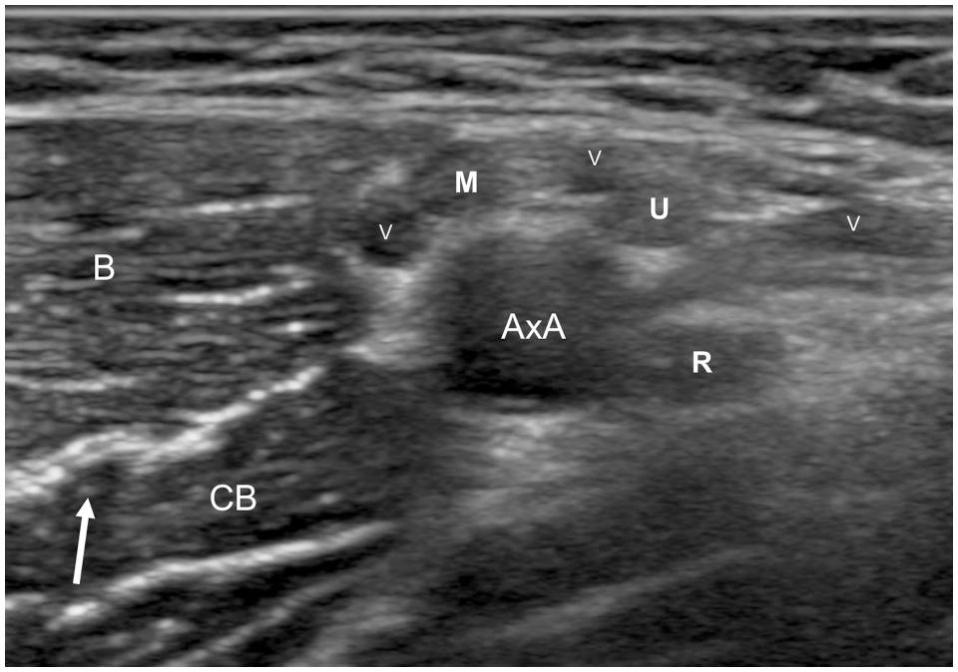
Transverse ultrasound of axillary fossa showing the musculocutaneous nerve (MCN) lying between the biceps (B) and coracobrachialis (Cb) muscles. The median nerve (M) consistently lies closest to the biceps muscle. The radial nerve (R) tends to lie towards the posterior of the axillary artery while the ulnar nerve lies superomedial to the axillary artery between the radial and medial nerves.

Then, fully abduct the arm to examine the axillary region. The brachial artery can be identified in the proximal arm and followed proximally in the transverse plane to the axilla, where the medial, ulnar, and radial nerves align around the axillary artery (Figure 7).

## Pathology

Common pathologies affecting the brachial plexus are:

- Irradiation plexopathy
- Nerve sheath tumours
- Traumatic injury
- Metastatic infiltration
- Thoracic outlet obstruction
- Neuralgic amyotrophy
- Inflammatory polyneuropathy

## Irradiation plexopathy

Irradiation-induced neural injury is a less common sequel of irradiation therapy nowadays with the use of tighter radiation fields, though this lower risk is somewhat offset by the increased usage of radiotherapy to treat tumours such as nasopharyngeal carcinoma, which were previously treated by surgery. If present, there is usually a 1- to 2-year latent period following radiotherapy before symptoms, such as pain and weakness, of brachial plexopathy arise.

**Figure 8.**
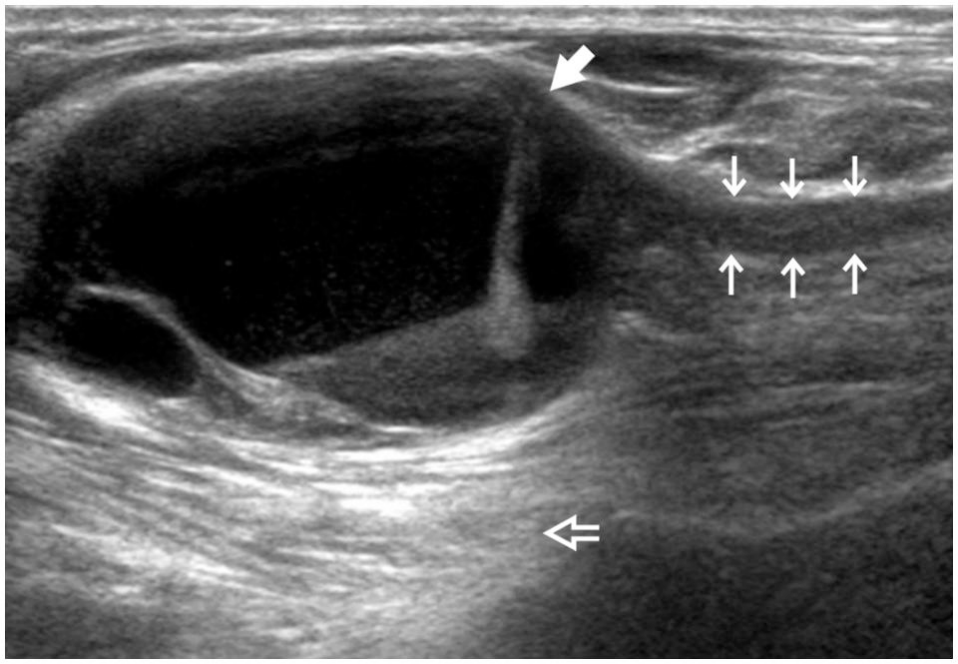
Longitudinal ultrasound showing a medium-sized nerve sheath tumour arising from the C5 root of the brachial plexus, which is in continuity with the tumour. The tumour (thick arrow) shows severe cystic degeneration with moderate posterior enhancement (open arrow).

The ultrasound appearances of brachial plexopathy are:

- increased size of the neural elements, particularly the upper roots, trunks, and divisions, as these are the most frequently affected components of the brachial plexus (Figure 8);
- poor delineation of neural components (Figure 8);
- obliterated or hypoechoic (“dirty”) perineural fat (Figure 8);
- clumping or adhesions of neural elements, particularly the divisions (Figure 8);
- neural or perineural hyperaemia;
- neural and perineural stiffness.

Irradiation plexopathy gradually transitions from normality to abnormality and vice versa rather than abruptly changing. An abrupt change favours metastatic infiltration. Management is mainly symptomatic. Omentoplasties and other vascularized flaps have been used, though they show no meaningful improvement in motor function. Nerve transfer may help to relieve neuropathic pain and improve motor function.^9^^,^^10^

## Tumours

**Figure 9.**
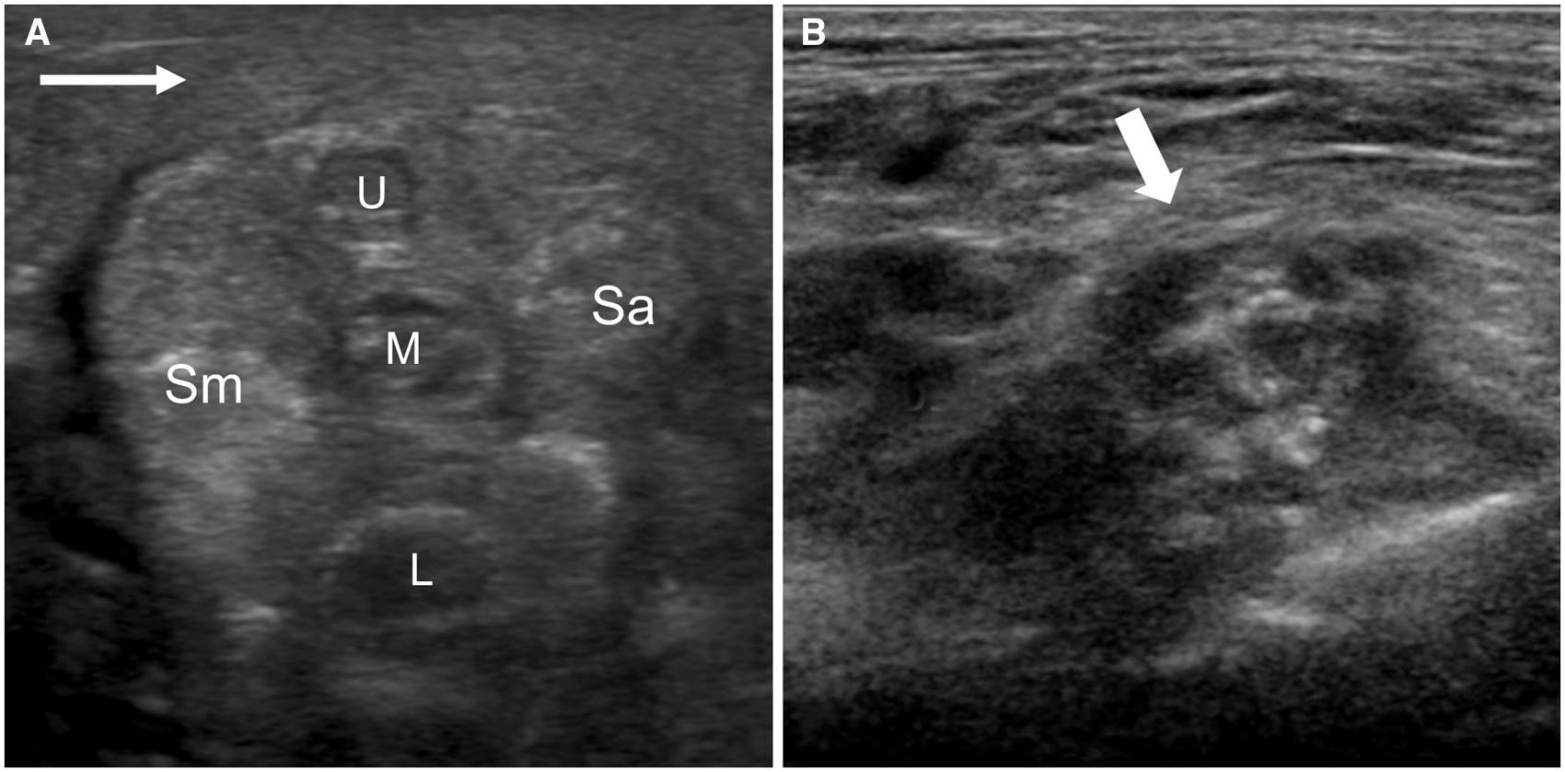
(A) Transverse ultrasound showing severe irradiation plexopathy of the interscalene triangle with perineural fat fibrosis leading to poor definition of the trunks (arrows) as well as severe dermal and subcutaneous thickening and hypoechogenicity due to fibrosis. (B) Transverse ultrasound showing moderate to severe irradiation plexopathy of the divisions, which are thickened, ill-defined, and clumped with hypoechogenicity of the adjacent fat due to fibrosis (arrows). Abbreviations: Sa = scalenus anterior muscle; Sm = scalenus medius muscle.

90% of brachial plexus tumours are nerve sheath tumours. 90% of these nerve sheath tumours are benign, and 90% of these benign nerve sheath tumours are schwannomas (Figure 9). The ultrasound appearances of brachial plexus nerve sheath tumours are similar to those occurring in peripheral nerves, ie, a hypoechoic mass with neural connectivity, posterior acoustic enhancement, and mild to moderate hyperaemia. Some tumours may have myxoid degeneration or haemorrhage. Others may be largely cystic (“cystic schwannoma”).

No ultrasound feature enables one to definitely distinguish between schwannoma and neurofibroma, though some features make schwannoma more likely. Malignant tumour should be suspected if the nerve sheath tumour is large, irregular, and rapidly growing clinically or on serial imaging studies. Tumour infiltration to the pleura, ribs, or vertebrae, though uncommon, is a clear indicator of malignancy. Percutaneous biopsy is very helpful if malignancy is suspected. Benign-looking nerve sheath tumours do not need biopsy for diagnosis.

## Metastases

Metastasis to the brachial plexus tends to be a feature of advanced malignancy, especially from breast and lung carcinoma. The trunks, divisions, and cords are more susceptible to metastatic spread, particularly the lower components. Symptoms tend to be of more rapid onset, more severe, and more painful than irradiation plexopathy.

**Figure 10.**
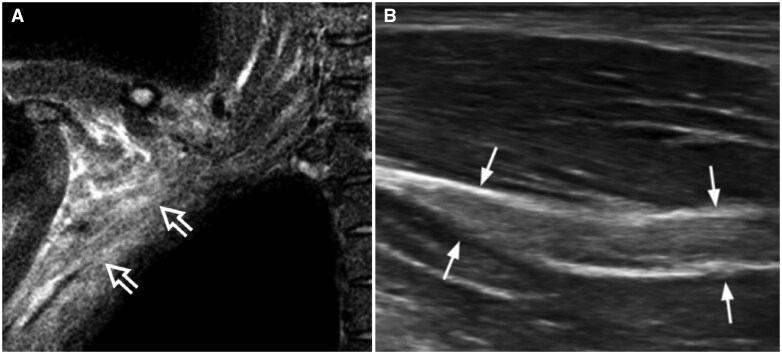
27-year-old man with arm weakness following traction injury. (A) T2-weighted fat-suppressed MR image shows severe oedema in the subcoracoid and axillary regions (open arrows) but no specific nerve injury. (B) Longitudinal ultrasound, on the following day, shows severe musculocutaneous nerve swelling (arrows) consistent with a severe traction injury.

Metastases are more likely if there is:

- a discrete mass. The more discrete the mass, the more likely it is metastatic (Figure 10);
- Hyperaemic mass. The more hyperaemic the mass, the more likely it is metastatic;
- involvement of the lower components of the brachial plexus;
- concurrent metastatic neck nodes;
- tumour infiltration to the pleura, ribs, or vertebrae is a clear indication of malignancy.

Occasionally, it may be difficult to distinguish scirrhous metastases arising from, for example, breast carcinoma, from nodular radiation fibrosis. Percutaneous biopsy can make this distinction, helping to confirm or refute metastatic disease. Even when the mass is inseparable from the brachial plexus, it is reasonable to still undertake biopsy, using an educated estimation as to where neural elements are likely to be located. The benefits of confirming the diagnosis usually outweigh the relatively low risk of neural injury.

## Trauma

The most common form of brachial plexus injury is traction injury, usually sustained during a road traffic accident. One needs to ascertain whether the injury is preganglionic (ie, injury to the nerve rootlets proximal to the dorsal root ganglion) or postganglionic injury (ie, injury to the ventral rami distal to the dorsal root ganglia). Postganglionic nerve root injury is amenable to surgical repair, while preganglionic injury is not. This distinction between pre- and postganglionic injury can only be made with MRI examination.

**Figure 11.**
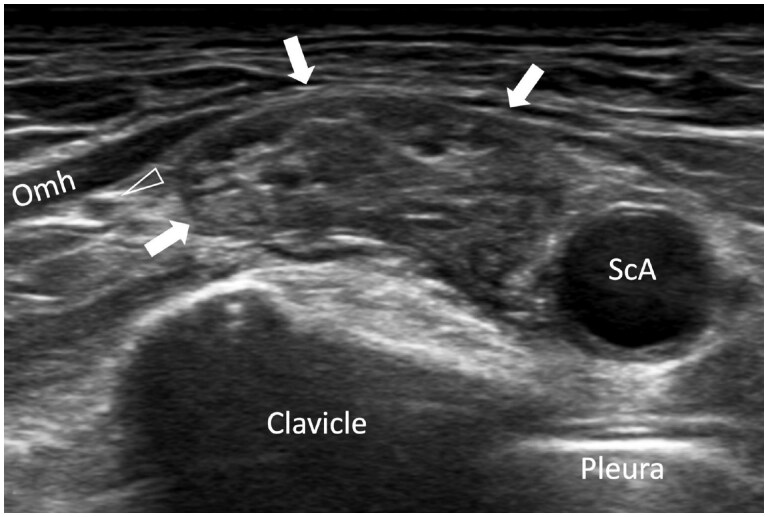
68-year-old man with known diffuse large B-cell lymphoma and arm pain for 2 months. Transverse US shows severe thickening of the soft tissues around the divisions (arrows) lateral to the subclavian artery (ScA) consistent with lymphoma infiltration. The suprascapular nerve deep to the omohyoid muscle (Omh) is not involved (arrowhead).

Once the intraspinal and intra-foraminal areas have been assessed with MRI, ultrasound is useful to examine the extra-foraminal components of the brachial plexus following trauma provided the patient is suitable (Figure 11). Ultrasound assessment may be limited if there has been severe trauma with upper thoracic fractures, soft tissue swelling, or an open wound. The patient also needs to be able to abduct the arm to fully examine the axillary region.

One should evaluate if there is any swelling, partial or complete transection, or compression of the neural elements. Longitudinal scanning of any abnormal area seen on transverse scanning is helpful. It is also helpful to reexamine the patient after a few weeks once features such as swelling and haematoma have settled, as abnormalities may become more apparent than on initial ultrasound examination. The role of ultrasound in birth-related brachial plexus injury is still not clear.

## Thoracic outlet syndrome

TOS usually results from dynamic, rather than static, compression of either the brachial plexus or the subclavian vessels and usually results from trauma, repetitive movements, or fibromuscular bands.^11^ Cadaveric dissection reveals that about two-thirds of the general population have some thoracic outlet variant, mostly anomalous fibromuscular bands.^12^ About 90% of TOS is due to neurogenic compression.^11^ For vascular compression, arterial compression is more common than venous compression. Suspected TOS is the single most difficult pathology of the brachial plexus to confidently diagnose and characterize with ultrasound. Increasing use is made of CT or MR angiography in neutral and abducted positions, but this primarily evaluates arterial compression and does not have the dynamic versatility of ultrasound.

Examination for TOS should begin with determining whether a cervical rib is present. Assessment of prior radiographs, CT, or MRI is helpful in this respect. Cervical ribs arise from the seventh cervical vertebra and are present in 2% of the general population.^13^ Most cervical ribs are asymptomatic. Cervical ribs can be categorized as either (1) extending just beyond the C7 transverse process, (2) almost touching the first rib, (3) associated with a fibrous band or cartilage attached to the first rib, and (4) completely fused to the first rib. On ultrasound, cervical ribs are seen protruding from the seventh cervical vertebra and may have an echopoor distal tip due to attached costal cartilage, resulting in an ultrasound appearance similar to a neonatal femoral head.^14^

About 90% of subjects have some abnormality of thoracic outlet anatomy on anatomical studies.^12^ Considerable anatomical variation exists with respect to the upper roots, the upper trunk, and the anterior scalene muscle (ASM). The classic “traffic light” pattern of 3 interscalene trunks is only seen in about one-third of normal subjects, with most having some upper trunk variation.^15^ Nearly all these anatomical variants are asymptomatic. The most common variant is C5 and C6 passing through the ASM (“superior piercing variant”). A less common variant is C5 passing anterior to the ASM (“C5 anterior variant”). Even less common variants are (a) C5 and C6 both passing anterior to the ASM, (b) C5 and C6 passing through a double ASM, and (c) C5 passing anterior and C6 passing through the ASM.^16^ The C5 and C6 roots may very occasionally be compressed as they penetrate the ASM, giving rise to upper plexus TOS symptoms. These anatomical variations are, however, not easy to define clearly on ultrasound, and there are no known imaging signs that differentiate a symptomatic from an asymptomatic anatomic variation.

**Figure 12.**
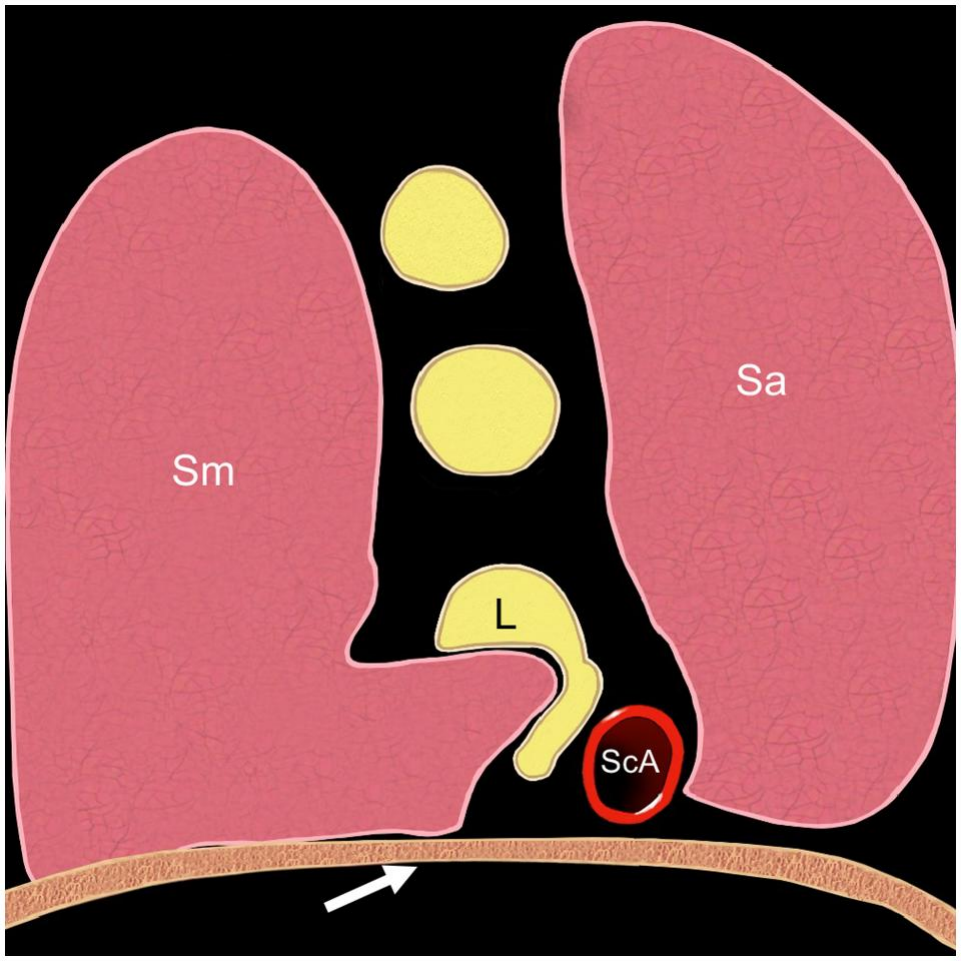
Schematic diagram showing indentation of lower trunk (L) (“wedge-sickle sign”), which can be caused by a fibromuscular band, a prominent or medially placed scalenus medius muscle, or a high-riding first rib (arrow). Abbreviations: Sa = scalenus anterior muscle; Sm = scalenus medius muscle; ScA = subclavian artery.

Swelling or indentation of the C7, C8, or T1 roots or the lower trunk is probably the commonest ultrasound sign of neurogenic TOS.^6^^,^^7^ Indentation of the lower roots or trunk may result from a cervical rib, a hyperechoic fibromuscular band, an atrophic scalenus minimus muscle, or a hypertrophied medially inserted scalenus medius muscle. This indentation can lead to the “wedge-sickle sign”^6–8^ (Figure 12). To summarize, for assessment of neurogenic TOS, special attention should be paid to the position of the upper roots with respect to the ASM and the lower roots and trunk with respect to any indentation from an anomalous rib, fibromuscular band, or muscle. All of these features are difficult to access in practice. Ultrasound-guided botulinum toxin injection to the scalene and pectoralis minor muscles may be beneficial either as a primary treatment for neurogenic TOS or as a prelude to first rib resection.^17^

Anatomical variation also exists with respect to the subclavian vessels and ASM, though normally the ASM separates the artery and vein at the base of the interscalene triangle. Compromised subclavian arterial or venous flow can be evaluated on spectral Doppler imaging. Both the calibre and flow of the subclavian artery and vein are assessed with the patient lying supine and sitting upright with the head turned to the contralateral side. Clear criteria for examination and diagnosis of arterial TOS have not been established.^11^ The subclavian artery and vein are examined with the shoulder in a neutral position and abducted to 90° or 120° (“abduction”) and 180° (“hyperabduction”). Measurements can also be made with the arm in the position that provokes symptoms. Measurements made with the arm in 30° hyperextension (“Halstead maneuver”) produce significant arterial spectral disturbance in one-third of normal subjects, including even occlusion, and are thus not recommended.^11^ Symptoms are also common in normal subjects during abduction and are not necessarily associated with arterial disturbance.^11^ 120° shoulder abduction without hyperextension may be the preferred abducted position, as this seems to produce the least false positive results.^11^

The artery is assessed longitudinally using an infraclavicular window with normal arterial flow being triphasic.^11^ Arterial compression is considered to be present if there is a decrease or loss of arterial flow (ie, “dynamic occlusion”) in abduction or hyperabduction compared to the neutral position, and/or if a biphasic or monophasic flow pattern develops with turbulence (ie, “dynamic stenosis”) often associated with, at least, 2-fold increase in peak systolic velocity.^11^^,^^18–22^ One may also look for arterial narrowing at the costoclavicular gap during abduction (“funnel sign”).^7^

The vein is assessed longitudinally using an infra- or supraclavicular window with normal venous flow being monophasic with both atrial pulsatility and respiratory phasicity. Venous compression is considered to be present if the vein is visibly compressed on abduction or hyperabduction, with more than 50% reduction in venous flow compared to the neutral position and loss of normal atrial pulsatility and respiratory phasicity.^18^

## Neuralgic amyotrophy

Neuralgic amyotrophy is a preferable term to “brachial neuritis” or “Parsonage-Turner syndrome” as the disease often manifests beyond the brachial plexus. It is an inflammatory condition of unknown aetiology which leads to both motor and sensory symptoms, particularly in the periscapular region, though also more peripherally in the upper limb. The suprascapular nerve is the most frequently affected nerve seen on ultrasound as focal thickening of this nerve. A suprascapular nerve CSA of >4.2 mm^2^ had a sensitivity of 86% and a specificity of 92% for neuralgic amyotrophy.^23^

Other extraplexal nerves that tend to be affected are the long thoracic, axillary, median, and radial nerves as well as the anterior and posterior interosseous nerves.^24^ While MRI is excellent at demonstrating atrophic and denervation changes in the parascapular and other muscles, ultrasound is particularly advantageous in allowing ready assessment of nerves beyond the brachial plexus. One should examine those nerves which are most likely to be affected based on the clinical picture. On ultrasound examination, one should follow any clinically suspected nerve on transverse scanning, looking for any neural swelling and any alteration in neural fascicular pattern. The ultrasound appearances of neuralgic amyotrophy are localized neural swelling ± fascicular entwinement ± hourglass constriction.^24^ If one sees neural swelling on transverse ultrasound scanning, longitudinal scanning should be performed to evaluate the presence of hourglass constriction. Hourglass constriction requires surgical intervention.

## Inflammatory neuropathy

Recognizing chronic immune-mediated peripheral inflammatory neuropathy is important, as targeted anti-inflammatory treatment can be provided. The 2 main types of inflammatory neuropathy are chronic inflammatory demyelinating neuropathy (CIDP) and multifocal motor neuropathy (MMN). CIDP is a sensory and motor neuropathy, while MMN is a predominant motor neuropathy. Both upper limbs tend to be affected.

The ultrasound features are similar for both and comprise nerve swelling, though neural hyperaemia may also be found. The brachial plexus and any clinically affected nerves should be examined. Trunk size >9 mm^2^ has been considered as abnormally large along with median nerve enlargement in the forearm >10 mm^2^ and upper arm >13 mm.^2^^,^^25^ The CSA of the C5-C7 roots and the median nerve were larger in CIDP than MMN patients, with measurements of the roots being preferable to the trunks, given the variability in trunk morphology.^26^

## Learning brachial plexus ultrasound, reporting, and when to do a follow-up MRI

As referrals for brachial plexus ultrasound examinations are generally low, one needs to build up a referral base. The best way to learn brachial plexus ultrasound is to examine normal volunteers or patients initially. Once familiar with the normal ultrasound appearances of the brachial plexus, consider asking patients attending for brachial plexus MRI, if they could come for a pre-MRI ultrasound. Most patients are happy to attend. Finally, liaise with clinicians to refer cases for brachial plexus ultrasound. The learning curve for assessment of the trunks, divisions, cords, and terminal branches is short. Confidence in assessing the roots takes longer. The suprascapular nerve is reasonably easy to identify proximally as it emerges from C5 or the upper trunk. It initially travels with the divisions but then separates from them and runs deep to the omohyoid muscles. Tracing the suprascapular nerve to the mid-clavicular line is usually sufficient. Assessment of the smaller nerves in the neck, such as dorsal scapular and long thoracic nerves, requires more experience.

The most common referral indication for brachial plexus ultrasound seems to be to exclude brachial plexus pathology in patients with non-specific arm pain and weakness. For these patients and other patients with normal brachial plexus examinations, a brief report such as “Unremarkable appearances of brachial plexus from the roots to the axilla” is given. For brachial plexopathy, grade the severity of perineural fibrosis as mild, moderate, or severe and state which parts of the brachial plexus are most affected.^27^ For suspected malignancy, give an indication as to how confident you are regarding the presence of metastatic infiltration and suggest biopsy, follow-up, or further work-up if unsure.

For suspected brachial plexopathy, nerve sheath tumour, metastatic infiltration, or assessment of extra-foraminal trauma, there is little or no additional benefit in performing MRI after brachial plexus ultrasound, as the likelihood of finding an overlooked abnormality is very low.^1^^,^^3^ For neuralgic amyotrophy, inflammatory neuropathy, or TOS, MRI is still recommended if brachial plexus ultrasound is normal.^1^^,^^3^

## Current limitation with brachial plexus ultrasound

The main limitations currently with brachial plexus ultrasound are:

- Recognizing mild radiation plexopathy. Ultrasound elastography may help in this regard, though it has not been tested.
- Recognizing mild inflammatory neuropathy. As neuropathy is a continual progressive disorder from mild to severe severity, using neural swelling as a sole criterion is always going to be limited. A combination of ultrasound and MRI with diffusion tensor imaging may prove more helpful than ultrasound alone in helping to firm up a clinical diagnosis of inflammatory polyneuropathy.
- Recognizing some cases of neuralgic amyotrophy. MRI generally is more sensitive than ultrasound, as MRI can recognize muscle oedema as an early feature of denervation with greater sensitivity than ultrasound. Potentially AI techniques may help in ultrasound recognition of muscle oedema.
- A clearer evaluation of the causes and ultrasound diagnostic criteria for neuralgic and vascular TOS is needed.
- Prospective studies to confirm that ultrasound is as sensitive as MRI in the detection of a wide spectrum of brachial plexus pathologies are needed. Retrospective studies have indicated this to be the case. Surgical confirmation is not necessary, as most brachial pathologies do not have surgical confirmation.
- Use of computational analytic techniques to confirm good side-to-side concordance in brachial plexus morphology would be helpful. Such a study could be performed with MRI rather than with ultrasound, as MRI planes are more standardized.

In summary, ultrasound is a very good method of evaluating the brachial plexus, though it tends to be underutilized in clinical practice. Ultrasound can be the first-line investigation in most patients with suspected brachial plexus pathology. If brachial plexus ultrasound is normal, the likelihood of finding an overlooked abnormality on MRI is low. Ultrasound is also helpful in evaluating neurological disorders that may concurrently involve extraplexal nerves. MRI has advantages in showing muscle denervation better than ultrasound and in enabling assessment of the intraspinal and intra-foraminal nerve roots relevant to the brachial plexus. Overall, both examinations are complimentary for brachial plexus assessment, though ultrasound examination alone will suffice in most cases.
